# Gut Dysbiosis and Hemodynamic Changes as Links of the Pathogenesis of Complications of Cirrhosis

**DOI:** 10.3390/microorganisms11092202

**Published:** 2023-08-31

**Authors:** Irina Efremova, Roman Maslennikov, Elena Poluektova, Maria Zharkova, Anna Kudryavtseva, George Krasnov, Maria Fedorova, Elena Shirokova, Evgenii Kozlov, Anna Levshina, Vladimir Ivashkin

**Affiliations:** 1 Department of Internal Medicine, Gastroenterology and Hepatology, Sechenov University, 119991 Moscow, Russiapolouektova@rambler.ru (E.P.); zharkovamaria@mail.ru (M.Z.); lewschinaa@yandex.ru (A.L.); kont07@yandex.ru (V.I.); 2 The Interregional Public Organization “Scientific Community for the Promotion of the Clinical Study of the Human Microbiome”, 119991 Moscow, Russia; 3 Consultative and Diagnostic Center No. 2, Moscow Health Department, 107564 Moscow, Russia; 4 Post-Genomic Research Laboratory, Engelhardt Institute of Molecular Biology of Russian Academy of Sciences, 119991 Moscow, Russiagskrasnov@mail.ru (G.K.); fedorowams@yandex.ru (M.F.); 5 Laboratory of Immunopathology, Department of Clinical Immunology and Allergy, Sechenov University, 119991 Moscow, Russia; kozlov-evgeny@bk.ru

**Keywords:** microbiota, microbiome, gut–liver axis, gut–heart axis, portal hypertension

## Abstract

The aim was to evaluate the relationship between gut dysbiosis and hemodynamic changes (hyperdynamic circulation) in cirrhosis, and between hemodynamic changes and complications of this disease. This study included 47 patients with cirrhosis. Stool microbiome was assessed using 16S rRNA gene sequencing. Echocardiography with a simultaneous assessment of blood pressure and heart rate was performed to assess systemic hemodynamics. Patients with hyperdynamic circulation had more severe cirrhosis, lower albumin, sodium and prothrombin levels, higher C-reactive protein, aspartate aminotransferase and total bilirubin levels, and higher incidences of portopulmonary hypertension, ascites, overt hepatic encephalopathy, hypoalbuminemia, hypoprothrombinemia, systemic inflammation, and severe hyperbilirubinemia than patients with normodynamic circulation. Patients with hyperdynamic circulation compared with those with normodynamic circulation had increased abundance of Proteobacteria, Enterobacteriaceae, Bacilli, Streptococcaceae, Lactobacillaceae, Fusobacteria, Micrococcaceae, Intestinobacter, Clostridium sensu stricto, Proteus and Rumicoccus, and decreased abundance of Bacteroidetes, Bacteroidaceae, Holdemanella, and Butyrivibrio. The systemic vascular resistance and cardiac output values correlated with the abundance of Proteobacteria, Enterobacteriaceae, Bacilli, Streptococcaceae, Lactobacillaceae, Micrococcaceae, and Fusobacteria. Heart rate and cardiac output value were negatively correlated with the abundance of Bacteroidetes. The mean pulmonary artery pressure value was positively correlated with the abundance of Proteobacteria and Micrococcaceae, and negatively with the abundance of Holdemanella.

## 1. Introduction

Hemodynamic changes in cirrhosis were described half a century ago and consist of arterial vasodilation (decreased systemic vascular resistance), hypotension, and increased cardiac output. This is defined as hyperdynamic circulation [1,2,3]. The study of experimental models of cirrhosis led to the hypothesis that these changes arise as a response to subclinical systemic inflammation. The penetration of gut bacteria into the lymph, liver, ascitic fluid, and portal and systemic circulation (bacterial translocation) in cirrhosis leads to the activation of the innate immune system by their pathogen-associated molecular patterns and causes the development of this systemic inflammatory response [1,2,3,4,5,6,7]. Experts suggest that hyperdynamic circulation aggravates portal hypertension, creating a predisposition for the development of various complications of cirrhosis [3,4,5,6,7]. The main factors contributing to bacterial translocation are increased intestinal barrier permeability, small intestinal bacterial overgrowth (SIBO), and changes in the composition of the gut microbiota (gut dysbiosis) [4]. The relationship between SIBO, hyperdynamic circulation, and complications of cirrhosis has been established [8,9]. However, despite the intensive study of gut dysbiosis, no previous research has described its effect on systemic hemodynamics in cirrhosis [10,11,12,13,14,15,16,17,18,19,20,21,22,23,24,25]. This study aimed to determine the relationship between gut dysbiosis and hemodynamic changes in cirrhosis as well as the relationship between these changes and the complications of this disease.

## 2. Materials and Methods

### 2.1. Patients

In this cross-sectional observational study, 100 consecutive patients with cirrhosis were admitted to the Department of Hepatology’s Clinic for Internal Diseases, Gastroenterology and Hepatology at Sechenov University (Moscow, Russia) and screened for inclusion. The study procedures were explained to potential participants, and written informed consent was obtained before enrollment. The present study was approved by the Ethics Committee of Sechenov University in accordance with the Declaration of Helsinki.

**Figure 1:**
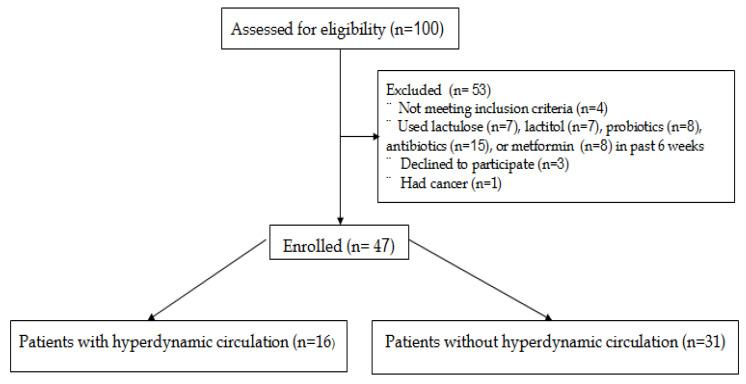
CONSORT 2010 flow diagram.

The inclusion criteria were as follows: diagnosis of cirrhosis and age between 18 and 70 years. The diagnosis of cirrhosis was established on the basis of presence of chronic liver disease, clinical and laboratory findings (presence of signs of portal hypertension and/or decreased liver function), and liver transient elastography data (>12 kPa) [26]. The exclusion criteria were as follows: use of lactulose, lactitol, or other prebiotics, probiotics, antibiotics, or metformin in the past 6 weeks; alcohol consumption in the past 6 weeks; or inflammatory bowel disease, cancer, or any other serious disease. Of the original 100 patients screened for inclusion, 47 met the criteria and were enrolled in the study (Figure 1).

### 2.2. Gut Microbiome Analysis

The morning after admission, a stool sample was taken into a sterile disposable container and immediately frozen at −80 °C [27].

DNA from the stool was isolated using the MagNa Pure Compact Nucleic Acid Isolation Kit I (Roche, Basel, Switzerland) according to the manufacturer’s instructions. Libraries for sequencing were prepared by two rounds of PCR amplification. In the first round, specific primers for the v3–v4 region of the 16S ribosomal RNA gene were used [28]:

16S-F TCGTCGGCAGCGTCAGATGTGTATAAGAGACAGCCTACGGGNGGCWGCAG and 16S-R GTCTCGTGGGCTCGGAGATGTGTATAAGAGACAGGACTACHVGGGTATCTAATCC.

After amplification, the PCR product was purified using AMPure XP magnetic beads (Beckman Coulter, Brea, CA, USA). Then, a second round of PCR was performed to attach specific adapters and enable multiplexing of the samples. To begin, 5 μL of the first PCR product was added to the reaction after ball cleaning with primers containing Illumina indices (Nextera XT Index v2 Primers; San Diego, CA, USA) and adapter sequences as well as 2× KAPA HiFi HotStart ReadyMix. The amplification products were also purified using AMPure XP beads (Beckman Coulter). The concentrations of the prepared libraries were then measured using a Qubit 2.0 fluorimeter (London, UK) and quantitative PCR. The quality of the libraries was assessed using the Agilent 2100 Bioanalyzer (Santa Clara, CA, USA). The libraries were mixed in equal proportions and diluted to the required concentration to be run on a MiSeq (Illumina) device. Paired-end readings of 300 + 300 nucleotides were obtained. Reads were trimmed from the 3′-tail with Trimmomatic (Illumina) and then merged into a single amplicon with the MeFiT tool [29,30]. We did not perform operational taxonomic unit picking; instead, we classified amplicon sequences with the Ribosomal Database Project (RDP) classifier and RDP database [31].

### 2.3. Systemic Hemodynamic Assessment

Echocardiography was performed at rest according to the guidelines of the American Society of Echocardiography [32,33,34,35]. The systolic and diastolic blood pressure and heart rate were measured using an automatic oscillometric sphygmomanometer (AND, Tokyo, Japan) simultaneously with the assessment of the stroke volume. Calculations of hemodynamic parameters are presented in Table 1 [32,33,34,35,36,37]. The criterion for portopulmonary hypertension was a combination of the presence of signs of portal hypertension and mean pulmonary artery pressure above 25 mm Hg [38].

No generally accepted criteria for hyperdynamic circulation are available. Therefore, we diagnosed a patient with this disorder if their cardiac output was greater than the mean + two standard deviations (5.5 L/min) of healthy individuals examined in the same way during the check-up [2,3,5,39]. The control group of healthy participants (n = 50) did not significantly differ from the patients with cirrhosis in terms of age and gender distribution (Table 2).

### 2.4. Statistical Analysis

Statistical analysis was performed with STATISTICA 10 (StatSoft Inc., Tulsa, OK, USA) and SPSS Statistics 26(IBM SPSS, Armonk, NY, USA) software. The data are presented as medians [interquartile ranges]. The abundance of taxa of the gut microbiota is presented as a percentage. Differences between continuous variables were assessed with the Mann–Whitney test because many variables were not distributed normally. Fisher’s exact test was used to assess the differences between categorical variables. Correlations between variables were computed using Spearman’s rank correlation.

An in-depth comparison of the gut microbiota between patients with hyperdynamic and normodynamic circulation was performed using the Linear discriminant analysis Effect Size (LEfSe; online resource “http://huttenhower.sph.harvard.edu/galaxy/ (accessed on 8 August 2023)” was used). The functional features of the gut microbiota in patients with hyperdynamic and normodynamic circulation were analyzed using the Phylogenetic Investigation of Communities by Reconstruction of Unobserved States (PICRUSt) method (the same online resource “http://huttenhower.sph.harvard.edu/galaxy/ (accessed on 8 August 2023)” was used).

*p*-values ≤ 0.05 were considered statistically significant.

## 3. Results

Hyperdynamic circulation was found in 16/47 (34.0%) patients, including 3/19 (18.8%) patients with Child–Pugh class A, 7/18 (44.4%) patients with Child–Pugh class B, and 6/10 (60.0% vs. 18.8%; *p* = 0.023) patients with Child–Pugh class C.

Cirrhosis patients with hyperdynamic circulation had higher values of left ventricle end-diastolic volume, stroke volume, cardiac output and mean pulmonary artery pressure and lower values of systemic vascular resistance than cirrhosis patients without hyperdynamic circulation (with normodynamic circulation) and normal controls. There was no significant difference in the above parameters between cirrhotic patients with normodynamic circulation and healthy controls. The ejection fraction was reduced in both groups of patients with cirrhosis compared with healthy controls, regardless of the type circulation. There was no significant difference between all groups in the values of heart rate and mean blood pressure (Table 2).

Patients with hyperdynamic circulation had more severe cirrhosis according to the Child–Pugh scale, lower albumin, sodium and prothrombin levels, higher C-reactive protein, aspartate aminotransferase and total bilirubin levels in the blood, and higher incidences of portopulmonary hypertension, ascites and clinically significant ascites (grade 2 and 3 ascites according to the classification of the International Club of Ascites), overt hepatic encephalopathy, hypoalbuminemia, hypoprothrombinemia, systemic inflammation, and severe hyperbilirubinemia than patients without hyperdynamic circulation. No significant difference between the groups of patients was observed for incidences of minimal ascites (grade 1 ascites according to the classification of the International Club of Ascites), mild hyperbilirubinemia, minimal hepatic encephalopathy, and esophageal varices, spleen size, main parameters of complete blood count, and serum creatinine, glucose and potassium levels (Table 3).

None of the included patients had spontaneous bacterial peritonitis or hepatopulmonary or hepatorenal syndrome.

There was no significant difference in alpha- and beta-diversity of the gut microbiota between patients with and without hyperdynamic circulation.

**Figure 2:**
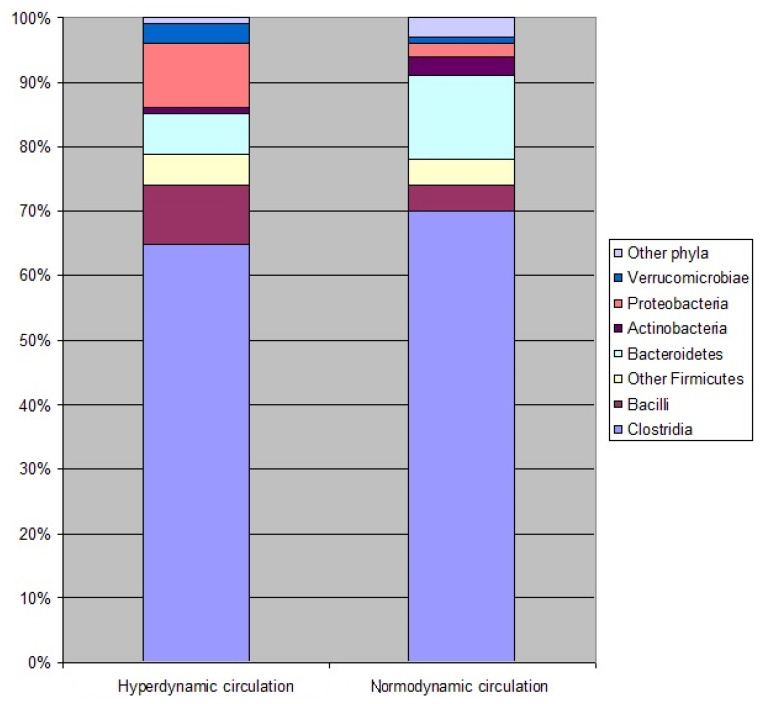
The composition of the gut microbiome in the cirrhosis patients with hyperdynamic and normodynamic circulation at the main taxa level.

Among the main gut microbiota taxa, the abundances of Proteobacteria, Enterobacteriaceae, Bacilli, Streptococcaceae, Lactobacillaceae, and Fusobacteria were increased and the abundance of Bacteroidetes was decreased in the gut microbiome of patients with hyperdynamic circulation compared to patients with normodynamic circulation (Table 4, Figure 2).

**Figure 3:**
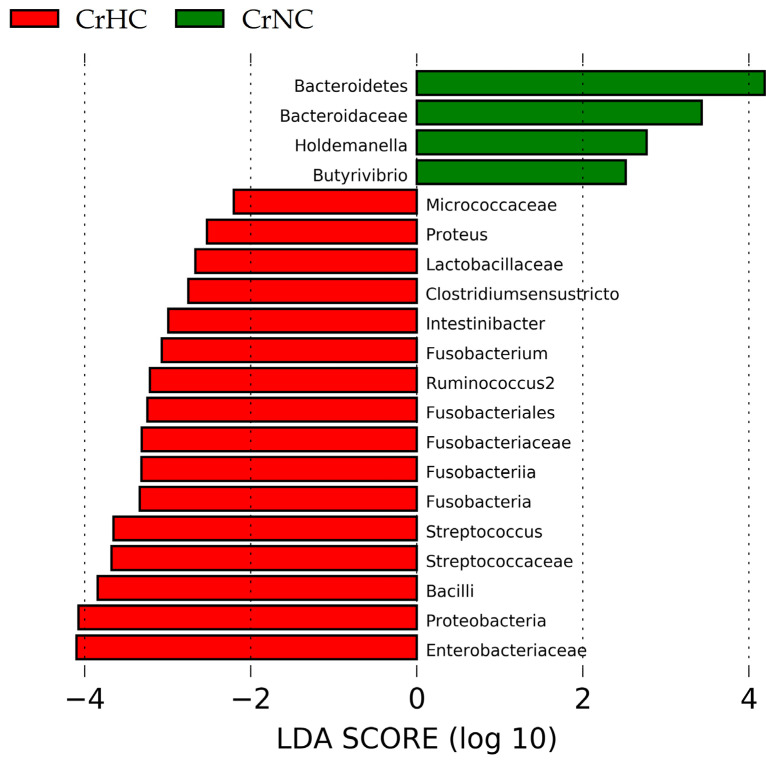
The gut microbiome taxa enriched in cirrhosis patients with hyperdynamic (CrHC) and normodynamic (CrNC) circulation.

**Figure 4:**
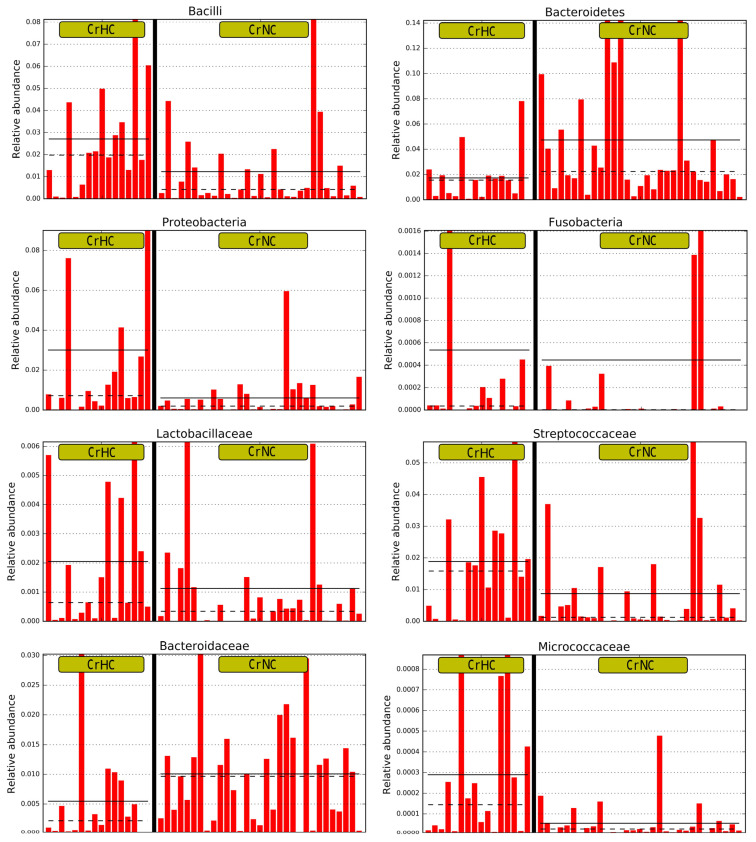
The main taxa enriched in cirrhosis patients with hyperdynamic (CrHC) and normodynamic (CrNC) circulation. Straight horizontal lines indicate the mean relative abundances; dotted horizontal lines show the medians of the relative abundances.

Linear discriminant analysis Effect Size (LEfSe) revealed that cirrhotic patients with hyperdynamic circulation, compared with patients with normodynamic circulation, had increased abundances of Proteobacteria (with Enterobacteriaceae family), Bacilli (with Streptococcaceae and Lactobacillaceae families), Fusobacteria (and subtaxa), Micrococcaceae, Intestinobacter, Clostridium sensu stricto, Proteus and Rumicoccus, and decreased abundances of Bacteroidetes (with Bacteroidaceae family), Holdemanella, and Butyrivibrio (Figure 3 and Figure 4).

**Figure 5:**
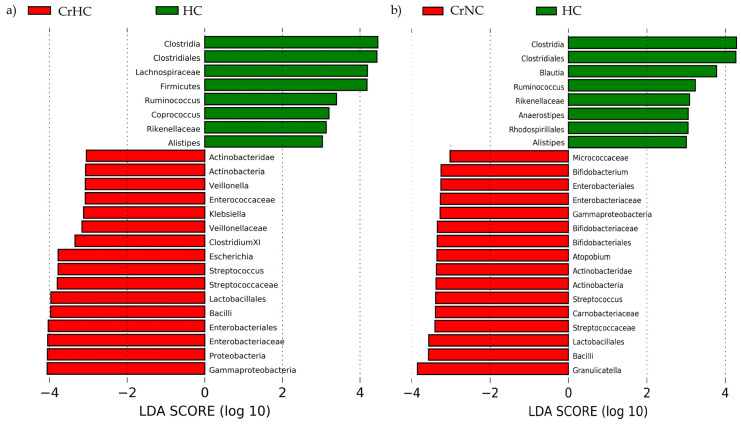
The gut microbiome taxa enriched in cirrhosis patients with (**a**) hyperdynamic (CrHC) and (**b**) normodynamic (CrNC) circulation compared with healthy controls (HC). Only differences with a log_10_ > 3.0 are presented.

Cirrhotic patients with hyperdynamic circulation had more pronounced gut dysbiosis than cirrhotic patients with normodynamic circulation when they were compared with healthy controls (Figure 5). In particular, the difference from healthy controls in the abundances of Bacilli, Streptococcaceae, Proteobacteria, and Enterobacteriaceae, whose growth in the gut microbiota was characteristic of cirrhosis [5], was higher in cirrhotic patients with hyperdynamic circulation than in cirrhotic patients with normodynamic circulation.

The systemic vascular resistance value negatively correlated with the abundance of Proteobacteria, Enterobacteriaceae, Bacilli, Streptococcaceae, Lactobacillaceae, Micrococcaceae, and Fusobacteria. Left ventricular end-diastolic volume positively correlated with the abundance of Proteobacteria, Enterobacteriaceae, Bacilli, Streptococcaceae, Micrococcaceae, and Fusobacteria. Heart rate was negatively correlated with the abundance of Bacteroidetes. The cardiac output value was positively correlated with the abundance of Proteobacteria, Enterobacteriaceae, Bacilli, Streptococcaceae, Lactobacillaceae, Micrococcaceae, and Fusobacteria, and negatively correlated with the abundance of Bacteroidetes. The mean pulmonary artery pressure value was positively correlated with the abundance of Proteobacteria and Micrococcaceae, and negatively with the abundance of Holdemanella. No correlation of the abundance of the main taxa of gut microbiome with mean blood pressure and ejection fraction values was observed (Table 5).

Phylogenetic Investigation of Communities by Reconstruction of Unobserved States revealed that gut microbiota of cirrhosis patients with hyperdynamic circulation had a higher abundance of genes involved in amino acid metabolism, bacterial invasion of epithelial cells, electron transfer, inorganic ion transport and metabolism, transcription related proteins, tryptophan metabolism, bacterial infection, and a lower abundance of genes involved in protein digestion and absorption (Table 6).

## 4. Discussion

Hyperdynamic circulation was observed in one-third of patients with cirrhosis, and the frequency of its detection increased with an increase in the Child–Pugh cirrhosis class. The increase in cardiac output was accompanied by a decrease in systemic vascular resistance, with no significant decrease in blood pressure. That is, the state of systemic hemodynamics in most of our patients was compensated by fluid retention and increased heart function, neutralizing the hypotonic effect of systemic vasodilation. The increased cardiac output was due to an increase in venous return to the heart, which led to an increase in end-diastolic volume. Heart rate and ejection fraction, which are other factors that could increase cardiac output, did not significantly differ between the groups of patients with and without hyperdynamic circulation, indicating their insignificant influence on its development. This is consistent with the underfilling hypothesis, which considers vasodilation as a primary disorder and fluid retention and increased venous return to the heart with an increase in cardiac output as secondary changes [2,3,4].

Notably, an increase in end-diastolic volume is usually characteristic of systolic heart failure, but it is not associated with a decrease in ejection fraction in patients with cirrhosis [9]. Moreover, the serum level of N-terminal brain natriuretic peptide, a biomarker of heart failure, does not depend on ejection fraction, but is associated, on the contrary, with increased heart function in these patients [40].

Complications of cirrhosis were differently associated with hyperdynamic circulation in our study. Some of them (hypoalbuminemia, hypoprothrombinemia, systemic inflammation, portopulmonary hypertension) were observed more often in patients with this disorder than in those without it. The presence of others (esophageal varices) was not associated with it. The association of hyperdynamic circulation with complications of cirrhosis from the third group (ascites, hyperbilirubinemia, hepatic encephalopathy) depended on their severity: it was absent in their mild and minimal forms, but their severe forms were associated with it. This may be considered as confirmation of the hypothesis that increased cardiac output aggravates the course of portal hypertension but is not its primary cause. Moreover, our study was cross-sectional and it is not entirely correct to judge causal relationships. The primary question here was whether decreased liver function led to the development of hyperdynamic circulation, whether hyperdynamic circulation worsened liver function, or whether they both exacerbated each other, leading to a vicious circle. Additional studies are required to determine the changes in liver function in patients with the same level of decreased liver function, depending on the presence or absence of hyperdynamic circulation. The incidence of hyperdynamic circulation development should be prospectively investigated and compared between patients with varying degrees of compensation for liver function in future studies.

Unfortunately, we could not measure the hepatic venous pressure gradient, which is considered the main quantitative characteristic of portal hypertension [41].

Our study is the first to assess the relationship between gut dysbiosis and hemodynamic changes in cirrhosis. Despite disagreements between the results of several previous studies, most indicated that the abundance of bacteria under the Proteobacteria phylum [10,11,12,13,14,15,16,17,18,21,22,23], which contains active endotoxin, and Bacilli class [13,14,15,16,17,18,19,20,21,22,23], which are capable of bacterial translocation, increases in the gut microbiome with cirrhosis. Thus, an increase in the abundance of these bacteria can be considered a biomarker of gut dysbiosis in cirrhosis. These bacteria are responsible for molecular (endotoxin) and cellular bacterial translocation in cirrhosis [42].

In this study, the abundance of Bacilli and Proteobacteria increased in patients with hyperdynamic circulation and correlated with the values of the main markers of hyperdynamic circulation, namely systemic vascular resistance and cardiac output. This may support the hypothesis that bacterial translocation of these bacteria and their components leads to vasodilation and hyperdynamic circulation. A similar relationship was also established for the minor taxon Fusobacteria, which also contain endotoxins. Only one article [22] reported an increase in the content of these bacteria in the gut microbiome in cirrhosis. This may be due to their low abundance in the gut microbiome, so these bacteria do not attract the attention of researchers.

An interesting finding was the decrease in Bacteroidetes abundance in patients with hyperdynamic circulation, considering these bacteria also have endotoxins. The abundance of these bacteria does not correlate with the degree of vasodilation but is associated with a decrease in heart rate, which can prevent the development of hyperdynamic circulation. The mechanism by which Bacteroidetes affect the heart rate is not clear. It seems that the presence of endotoxin is not an indicator of bacterial pathogenicity and ability to translocate. It should be remembered that Bacteroidetes, together with bacteria under the Clostridia class, are the main taxa of normal human microbiota, and changes in their abundance in cirrhosis compared with healthy individuals are reported differently in different publications. Bacteroidetes abundance either increases [11,24], decreases [10,19,22], does not change [21], or changes depending on the state of liver function [16] in cirrhosis. Bacteroidetes showed a protective effect against hyperdynamic circulation in our study.

The abundance of beneficial bacteria under the Clostridia class in the gut microbiome does not significantly differ between patients with and without hyperdynamic circulation and does not correlate with any of the hemodynamic parameters in cirrhosis.

Changes in the gut microbiome in hemodynamic circulation mainly signify a redistribution of the proportion of bacteria containing endotoxins, where Proteobacteria and Fusobacteria that have active endotoxins replace Bacteroidetes that have weak endotoxins [43] (Figure 2).

The role of minor taxa of the gut microbiota, which were associated with hemodynamic changes in cirrhosis in this study, remains to be established.

Phylogenetic Investigation of Communities by Reconstruction of Unobserved States found that the gut microbiota in cirrhotic patients with hyperdynamic circulation, compared with the gut microbiota in cirrhotic patients with normodynamic circulation, has a greater abundance of the genes that make it more metabolically active (the genes of amino acid metabolism, electron and inorganic ion transport, gene transcription) and aggressive (the genes of factors of bacterial invasion), and this predisposed to the development of bacterial translocation, provoking an inflammatory vasodilatory response to it.

Probiotics, which are living bacteria used for dysbiosis, showed their effects on hemodynamic parameters in cirrhosis in small uncontrolled studies, but require randomized controlled trials to confirm [44].

Unfortunately, we were unable to determine the levels either of blood lipopolysaccharide or short-chain fatty acids in feces. These remain tasks for future research.

Our study is the first to confirm that gut dysbiosis is associated with hemodynamic changes in cirrhosis. We further showed that the presence of these changes is associated with a number of complications of cirrhosis. Thus, hemodynamic changes may be considered a pathogenetic link between gut dysbiosis and these complications of cirrhosis. However, this hypothesis requires verification in further prospective studies, the ideas of which we also proposed. All of these contribute to the strength of our study.

A limitation of our study is its small sample size, although this did not prevent us from obtaining significant results. Another limitation of our study was that we analyzed only the fecal microbiome, despite the fact that the mucosal microbiota of the parietal mucus of the colon may exert more effects on the human body than the luminal microbiota. It should also be taken into account that after leaving the body, the intestinal contents are transferred from anaerobic to aerobic conditions; therefore, facultative anaerobes continue to multiply, and obligate anaerobes stop multiplying in the feces. This leads to the fact that the composition of the fecal microbiota becomes not identical to the composition of the luminal gut microbiota. Although we froze fecal samples almost immediately after the patients had defecated, this difference cannot be completely ruled out.

## 5. Conclusions

In conclusion, we have shown that gut dysbiosis is associated with hyperdynamic circulation, which in turn is associated with a number of complications of cirrhosis.

## Figures and Tables

**Table 1:** Calculations of hemodynamic parameters.

Parameter	Calculation
End-diastolic and end-systolic volume of the left ventricle	Modified Simpson’s disk method
Ejection fraction of the left ventricle	((end-diastolic volume) − (end-systolic volume))/(end-diastolic volume)
Stroke volume	(Doppler velocity time integral) × (cross-sectional aorta area) [36]
Mean arterial pressure	((systolic blood pressure) + 2 × (diastolic blood pressure))/3
Cardiac output	(stroke volume) × (heart rate)
Systemic vascular resistance	(mean arterial pressure)/(cardiac output)
Systolic pulmonary artery pressure	(right atrium pressure estimated from diameter of inferior vena cava and respiratory changes) + 4 × (the peak velocity of the tricuspid valve regurgitant jet)^2^ [34,35]
Mean pulmonary artery pressure	0.61 × (systolic pulmonary artery pressure) + 2 mmHg [37]

**Table 2:** Main and hemodynamic parameters of cirrhotic patients with hyperdynamic and normodynamic circulation, as well as healthy controls.

	Cirrhosis with Hyperdynamic Circulation (CrHC) (n = 16)	Cirrhosis with Normodynamic Circulation (CrNC) (n = 31)	Healthy Controls (HC) (n = 50)	*p*, CrHC vs. CrNC	*p*, CrHC vs. HC	*p*, CrNC vs. HC
Age, years	49 [39–53]	53 [37–60]	46 [42–52]	0.432	0.887	0.061
Body mass index, kg/m^2^	24.1 [22.5–27.2]	24.1 [22.7–27.6]	25.7 [23.9–27.3]	0.937	0.262	0.125
Male/female	8/8	14/17	20/30	0.497	0.338	0.410
End-diastolic volume of the left ventricle, mL	129 [118–138]	97 [87–111]	98 [91–103]	<0.001	<0.001	0.782
Ejection fraction of the left ventricle, %	62.8 [61.2–64.4]	60.8 [58.6–63.8]	65.1 [62.5–66.3]	0.119	0.023	<0.001
Stroke volume, mL	79 [73–86]	60 [55–71]	62 [59–68]	<0.001	<0.001	0.248
Heart rate, bpm	77 [70–87]	70 [64–80]	73 [70–76]	0.092	0.103	0.509
Cardiac output, L/min	5.8 [5.6–6.7]	4.2 [3.8–4.6]	4.5 [4.2–4.8]	<0.001	<0.001	0.063
Mean blood pressure, mmHg	89 [77–92]	88 [83–95]	90 [87–92]	0.613	0.246	0.406
Systemic vascular resistance, dyn·s·cm^−5^	1096 [1011–1327]	1683 [1436–1917]	1633 [1484–1730]	<0.001	<0.001	0.315
Mean pulmonary artery pressure, mmHg	23.4 [17.6–26.4]	17.3 [13.6–20.3]	15.2 [13.7–16.8]	0.024	<0.001	0.106

**Table 3:** Main indicators of patients with cirrhosis with hyperdynamic and normodynamic circulation.

	Cirrhosis with Hyperdynamic Circulation (n = 16)	Cirrhosis with Normodynamic Circulation (n = 31)	*p*
Etiology of cirrhosis: alcohol	6 (37.5%)	7 (22.6%)	>0.050
autoimmune hepatitis	2 (12.5%)	5 (16.2%)	
HBV	1 (6.3%)	5 (16.1%)	
HCV	2 (12.5%)	7 (22.6%)	
mixed	4 (25.0%)	5 (16.1%)	
cryptogenic	1 (6.3%)	2 (6.5%)	
Child–Pugh score	9 [8–11]	6 [6–8]	0.007
Esophageal varices (Grade 1), n (%)	4 (25.0%)	11 (35.5%)	0.349
Esophageal varices (Grade 2–3), n (%)	6 (37.5%)	17 (54.9%)	0.207
Minimal hepatic encephalopathy, n (%)	4 (25.0%)	14 (45.2%)	0.151
Overt hepatic encephalopathy, n (%)	9 (56.3%)	8 (23.8%)	0.042
Ascites, n (%)	12 (75.0%)	14 (45.2%)	0.049
Minimal ascites, n (%)	4 (25.0%)	9 (29.0%)	0.527
Clinically significant ascites, n (%)	8 (50.0%)	5 (16.1%)	0.018
Portopulmonary hypertension, n (%)	7 (43.8%)	5 (16.1%)	0.046
Red blood cells, 10^12^ cell/L	3.7 [3.1–4.2]	3.9 [3.7–4.5]	0.062
White blood cells, 10^9^ cell/L	4.5 [3.5–7.3]	3.5 [2.8–4.8]	0.106
Platelets, 10^9^ cell/L	89 [59–107]	79 [58–115]	0.866
Serum total protein, g/L	69 [63–75]	73 [63–78]	0.340
Serum albumin, g/L	31 [28–36]	38 [32–42]	0.014
Hypoalbuminemia (serum albumin < 35 g/L), n (%)	11 (68.8%)	9 (29.0%)	0.011
Serum total bilirubin, μmol/L	56 [36–83]	31 [22–51]	0.014
Mild hyperbilirubinemia (total bilirubin = 22–51 μmol/L), n (%)	5 (31.3%)	17 (54.8%)	0.110
Severe hyperbilirubinemia (total bilirubin > 51 μmol/L), n (%)	9 (56.3%)	7 (22.6%)	0.025
Prothrombin index (Quick test), %	53 [45–65]	66 [57–71]	0.003
Hypoprothrombinemia (prothrombin index < 60%), n (%)	10 (62.5%)	10 (32.3%)	0.047
Creatinine, mg/dL	0.8 [0.6–0.9]	0.7 [0.6–0.9]	0.694
Serum sodium, mmol/L	139 [138–141]	142 [140–144]	0.013
Serum potassium, mmol/L	4.2 [3.2–4.6]	4.3 [4.1–4.7]	0.121
Serum glucose, mmol/L	5.3 [4.7–5.6]	5.2 [4.6–5.7]	0.955
Alanine aminotransferase, U/L	58 [27–127]	36 [23–59]	0.167
Aspartate aminotransferase, U/L	77 [47–176]	47 [29–64]	0.022
Gamma glutamyl transferase, U/L	96 [37–144]	59 [27–140]	0.452
Alkaline phosphatase, U/L	235 [176–339]	214 [166–286]	0.598
C-reactive protein, mg/L	11.6 [5.3–17.1]	3.4 [0.8–9.8]	0.021
Systemic inflammation (C-reactive protein > 10 mg/L), n (%)	9 (56.3%)	6 (19.4%)	0.013
Splenic length, cm	15.7 [14.2–17.0]	15.7 [13.9–19.2]	0.788

**Table 4:** Comparison of the gut microbiome at different taxonomic levels between patients with hyperdynamic and normodynamic circulation (relative abundance, %).

Taxa	Cirrhosis with Hyperdynamic Circulation (n = 16)	Cirrhosis with Normodynamic Circulation (n = 31)	*p*
Firmicutes	87.2 [68.4–91.1]	84.8 [69.3–91.0]	0.745
Clostridia	71.4 [52.6–85.4]	76.0 [62.5–83.1]	0.711
Lachnospiraceae	34.0 [22.8–45.9]	36.5 [21.8–48.9]	0.745
Ruminococcaceae	24.3 [10.8–34.1]	22.2 [14.9–36.6]	0.745
Peptostreptococcaceae	1.3 [0.2–4.0]	0.2 [0.0–0.9]	0.071
Bacilli	6.1 [1.3–13.6]	1.1 [0.5–4.6]	0.042
Streptococcaceae	1.3 [0.3–8.1]	0.4 [0.1–3.1]	0.049
Lactobacillaceae	0.2 [0.2–1.2]	0.1 [0.0–0.4]	0.047
Negativicutes	0.5 [0.2–0.8]	0.4 [0.1–1.3]	0.884
Veillonellaceae	0.4 [0.1–0.8]	0.2 [0.0–0.8]	0.522
Erysipelotrichia	0.3 [0.1–0.5]	0.7 [0.3–1.1]	0.063
Holdemanella	0.03 [0.00–0.37]	0.00 [0.00–0.02]	0.022
Fusobacteria	0.0 [0.0–0.1]	0.0 [0.0–0.0]	0.047
Bacteroidetes	5.6 [0.9–6.4]	6.9 [4.5–15.7]	0.038
Bacteroidaceae	0.8 [0.1–2.4]	3.1 [0.8–4.2]	0.029
Rikenellaceae	0.1 [0.0–0.9]	0.4 [0.0–0.9]	0.278
Porphyromonadaceae	0.3 [0.0–0.4]	0.3 [0.1–0.5]	0.222
Prevotellaceae	0.3 [0.0–2.7]	0.3 [0.0–7.0]	0.493
Actinobacteria	0.9 [0.5–2.1]	0.7 [0.2–2.3]	0.400
Bifidobacteriaceae	0.5 [0.2–1.8]	0.4 [0.0–1.7]	0.479
Micrococcaceae	0.01 [0.00–0.02]	0.05 [0.01–0.11]	0.013
Proteobacteria	2.3 [1.2–8.2]	0.7 [0.1–2.2]	0.020
Enterobacteriaceae	2.0 [0.6–7.6]	0.3 [0.0–1.7]	0.008
Verrucomicrobiae	0.0 [0.0–0.2]	0.0 [0.0–1.5]	0.955
Akkermansiaceae	0.0 [0.0–0.2]	0.0 [0.0–0.9]	0.767

**Table 5:** Correlation matrix of the main taxa of the gut microbiome and the main hemodynamic parameters in cirrhosis.

	EDV	EF	SV	HR	CO	MBP	SVR	MPAP
Clostridia	N.S.	N.S.	N.S.	N.S.	N.S.	N.S.	N.S.	N.S.
Bacilli	r = 0.317; *p* = 0.029	N.S.	r = 0.330; *p* = 0.024	N.S.	r = 0.402; *p* = 0.005	N.S.	r = −0.328; *p* = 0.025	N.S.
Streptococcaceae	r = 0.406; *p* = 0.005	N.S.	r = 0.378; *p* = 0.009	N.S.	r = 0.415; *p* = 0.004	N.S.	r = −0.290; *p* = 0.048	N.S.
Lactobacillaceae	N.S.	N.S.	N.S.	N.S.	r = 0.373; *p* = 0.010	N.S.	r = −0.333; *p* = 0.022	N.S.
Fusobacteria	r = 0.311; *p* = 0.037	N.S.	N.S.	N.S.	r = 0.316; *p* = 0.034	N.S.	r = −0.424; *p* = 0.004	N.S.
Bacteroidetes	N.S.	N.S.	N.S.	r = −0.483; *p* = 0.001	r = −0.290; *p* = 0.048	N.S.	N.S.	N.S.
Proteobacteria	r = 0.454; *p* = 0.001	N.S.	r = 0.431; *p* = 0.002	N.S.	r = 0.435; *p* = 0.002	N.S.	r = −0.434; *p* = 0.002	r = 0.290; *p* = 0.048
Enterobacteriaceae	r = 0.447; *p* = 0.002	N.S.	r = 0.426; *p* = 0.003	N.S.	r = 0.479; *p* = 0.001	N.S.	r = −0.437; *p* = 0.002	N.S.
Micrococcaceae	r = 0.321; *p* = 0.031	N.S.	r = 0.360; *p* = 0.015	N.S.	r = 0.363; *p* = 0.014	N.S.	r = −0.362; *p* = 0.015	r = 0.339; *p* = 0.028
Holdemanella	N.S.	N.S.	N.S.	N.S.	N.S.	N.S.	N.S.	r = −0.356; *p* = 0.016

CO—Cardiac output; EDV—End-diastolic volume of the left ventricle; EF—Ejection fraction of the left ventricle; HR—Heart rate; MBP—Mean blood pressure; MPAP—Mean pulmonary artery pressure; N.S.—Not significant; SV—Stroke volume; SVR—Systemic vascular resistance.

**Table 6:** Significant differences in the functional properties of the gut microbiota in cirrhotic patients with hyperdynamic (CrHC) and normodynamic (CrNC) circulation.

Function of the Gut Microbiota	LOG (CrHC/CrNC)	*p*
Amino acid metabolism	0.08	0.043
Aminobenzoate degradation	0.08	0.043
Bacterial invasion of epithelial cells	0.53	0.019
Benzoate degradation	0.12	0.036
Dioxin degradation	0.15	0.027
Electron transfer carriers	0.44	0.004
Inorganic ion transport and metabolism	0.08	0.040
Limonene and pinene degradation	0.11	0.040
Lysine degradation	0.11	0.045
Naphthalene degradation	0.04	0.025
Phosphotransferase system (PTS)	0.19	0.020
Protein digestion and absorption	−0.29	0.036
Staphylococcus aureus infection	0.32	0.002
Stilbenoid, diarylheptanoid and gingerol biosynthesis	1.15	0.023
Transcription-related proteins	0.26	0.048
Tryptophan metabolism	0.11	0.045
Xylene degradation	0.12	0.030

## Data Availability

The datasets may be available from the corresponding author on reasonable request.

## References

[1] Di Pascoli M., Sacerdoti D., Pontisso P., Angeli P., Bolognesi M. (2017). Molecular Mechanisms Leading to Splanchnic Vasodilation in Liver Cirrhosis. J. Vasc. Res..

[2] Moller S., Bendtsen F. (2018). The pathophysiology of arterial vasodilatation and hyperdynamic circulation in cirrhosis. Liver Int..

[3] Bolognesi M., Di Pascoli M., Verardo A., Gatta A. (2014). Splanchnic vasodilation and hyperdynamic circulatory syndrome in cirrhosis. World J. Gastroenterol..

[4] Ponziani F.R., Zocco M.A., Cerrito L., Gasbarrini A., Pompili M. (2018). Bacterial translocation in patients with liver cirrhosis: Physiology, clinical consequences, and practical implications. Expert. Rev. Gastroenterol. Hepatol..

[5] Maslennikov R., Ivashkin V., Efremova I., Poluektova E., Shirokova E. (2021). The gut-liver axis in cirrhosis: Are hemodynamic changes a missing link?. World J. Clin. Cases.

[6] Gómez-Hurtado I., Such J., Sanz Y., Francés R. (2014). Gut microbiota-related complications in cirrhosis. World J. Gastroenterol..

[7] Simbrunner B., Mandorfer M., Trauner M., Reiberger T. (2019). Gut-liver axis signaling in portal hypertension. World J. Gastroenterol..

[8] Maslennikov R., Pavlov C., Ivashkin V. (2018). Small intestinal bacterial overgrowth in cirrhosis: Systematic review and meta-analysis. Hepatol. Int..

[9] Maslennikov R., Pavlov C., Ivashkin V. (2019). Is small intestinal bacterial overgrowth a cause of hyperdynamic circulation in cirrhosis?. Turk. J. Gastroenterol..

[10] Jin M., Kalainy S., Baskota N., Chiang D., Deehan E.C., McDougall C., Tandon P., Martínez I., Cervera C., Walter J. (2019). Faecal microbiota from patients with cirrhosis has a low capacity to ferment non-digestible carbohydrates into short-chain fatty acids. Liver Int..

[11] Zeng Y., Chen S., Fu Y., Wu W., Chen T., Chen J., Yang B., Ou Q. (2020). Gut microbiota dysbiosis in patients with hepatitis B virus-induced chronic liver disease covering chronic hepatitis, liver cirrhosis and hepatocellular carcinoma. J. Viral Hepat..

[12] Kajihara M., Koido S., Kanai T., Ito Z., Matsumoto Y., Takakura K., Saruta M., Kato K., Odamaki T., Xiao J. (2019). Characterisation of blood microbiota in patients with liver cirrhosis. Eur. J. Gastroenterol. Hepatol..

[13] Chen Z., Xie Y., Zhou F., Zhang B., Wu J., Yang L., Xu S., Stedtfeld R., Chen Q., Liu J. (2020). Featured Gut Microbiomes Associated With the Progression of Chronic Hepatitis B Disease. Front. Microbiol..

[14] Zheng R., Wang G., Pang Z., Ran N., Gu Y., Guan X., Yuan Y., Zuo X., Pan H., Zheng J. (2020). Liver cirrhosis contributes to the disorder of gut microbiota in patients with hepatocellular carcinoma. Cancer Med..

[15] Lapidot Y., Amir A., Nosenko R., Uzan-Yulzari A., Veitsman E., Cohen-Ezra O., Davidov Y., Weiss P., Bradichevski T., Segev S. (2020). Alterations in the Gut Microbiome in the Progression of Cirrhosis to Hepatocellular Carcinoma. mSystems.

[16] Bajaj J.S., Heuman D.M., Hylemon P.B., Sanyal A.J., White M.B., Monteith P., Noble N.A., Unser A.B., Daita K., Fisher A.R. (2014). Altered profile of human gut microbiome is associated with cirrhosis and its complications. J. Hepatol..

[17] Bajaj J.S., Betrapally N.S., Hylemon P.B., Heuman D.M., Daita K., White M.B., Unser A., Thacker L.R., Sanyal A.J., Kang D.J. (2015). Salivary microbiota reflects changes in gut microbiota in cirrhosis with hepatic encephalopathy. Hepatology.

[18] Ahluwalia V., Betrapally N.S., Hylemon P.B., White M.B., Gillevet P.M., Unser A.B., Fagan A., Daita K., Heuman D.M., Zhou H. (2016). Impaired Gut-Liver-Brain Axis in Patients with Cirrhosis. Sci. Rep..

[19] Liu Y., Jin Y., Li J., Zhao L., Li Z., Xu J., Zhao F., Feng J., Chen H., Fang C. (2018). Small Bowel Transit and Altered Gut Microbiota in Patients With Liver Cirrhosis. Front. Physiol..

[20] Inoue T., Nakayama J., Moriya K., Kawaratani H., Momoda R., Ito K., Iio E., Nojiri S., Fujiwara K., Yoneda M. (2018). Gut Dysbiosis Associated With Hepatitis C Virus Infection. Clin. Infect. Dis..

[21] Maslennikov R., Ivashkin V., Efremova I., Alieva A., Kashuh E., Tsvetaeva E., Poluektova E., Shirokova E., Ivashkin K. (2021). Gut dysbiosis is associated with poorer long-term prognosis in cirrhosis. World J. Hepatol..

[22] Chen Y., Yang F., Lu H., Wang B., Chen Y., Lei D., Wang Y., Zhu B., Li L. (2011). Characterization of fecal microbial communities in patients with liver cirrhosis. Hepatology.

[23] Kakiyama G., Pandak W.M., Gillevet P.M., Hylemon P.B., Heuman D.M., Daita K., Takei H., Muto A., Nittono H., Ridlon J.M. (2013). Modulation of the fecal bile acid profile by gut microbiota in cirrhosis. J. Hepatol..

[24] Bajaj J.S., Vargas H.E., Reddy K.R., Lai J.C., O’Leary J.G., Tandon P., Wong F., Mitrani R., White M.B., Kelly M. (2019). Association Between Intestinal Microbiota Collected at Hospital Admission and Outcomes of Patients With Cirrhosis. Clin. Gastroenterol. Hepatol..

[25] Zhang L., Wu Y.N., Chen T., Ren C.H., Li X., Liu G.X. (2019). Relationship between intestinal microbial dysbiosis and primary liver cancer. Hepatobiliary Pancreat. Dis. Int..

[26] Berzigotti A., Tsochatzis E., Boursier J., Castera L., Cazzagon N., Friedrich-Rust M., Petta S., Thiele M. (2021). EASL Clinical Practice Guidelines on non-invasive tests for evaluation of liver disease severity and prognosis—2021 update. J. Hepatol..

[27] Fouhy F., Deane J., Rea M.C., O’Sullivan Ó., Ross R.P., O’Callaghan G., Plant B.J., Stanton C. (2015). The effects of freezing on faecal microbiota as determined using MiSeq sequencing and culture-based investigations. PLoS ONE.

[28] Zheng W., Tsompana M., Ruscitto A., Sharma A., Genco R., Sun Y., Buck M.J. (2015). An accurate and efficient experimental approach for characterization of the complex oral microbiota. Microbiome.

[29] Bolger A.M., Lohse M., Usadel B. (2014). Trimmomatic: A flexible trimmer for Illumina sequence data. Bioinformatics.

[30] Parikh H.I., Koparde V.N., Bradley S.P., Buck G.A., Sheth N.U. (2016). MeFiT: Merging and filtering tool for illumina paired-end reads for 16S rRNA amplicon sequencing. BMC Bioinform..

[31] Wang Q., Garrity G.M., Tiedje J.M., Cole J.R. (2007). Naive Bayesian Classifier for Rapid Assignment of rRNA Sequences into the New Bacterial Taxonomy. Appl. Environ. Microbiol..

[32] Lang R.M., Badano L.P., Mor-Avi V., Afilalo J., Armstrong A., Ernande L., Flachskampf F.A., Foster E., Goldstein S.A., Kuznetsova T. (2015). Recommendations for cardiac chamber quantification by echocardiography in adults: An update from the American Society of Echocardiography and the European Association of Cardiovascular Imaging. J. Am. Soc. Echocardiogr..

[33] Marwick T.H., Gillebert T.C., Aurigemma G., Chirinos J., Derumeaux G., Galderisi M., Gottdiener J., Haluska B., Ofili E., Segers P. (2015). Recommendations on the Use of Echocardiography in Adult Hypertension: A Report from the European Association of Cardiovascular Imaging (EACVI) and the American Society of Echocardiography (ASE). J. Am. Soc. Echocardiogr..

[34] Rudski L.G., Lai W.W., Afilalo J., Hua L., Handschumacher M.D., Chandrasekaran K., Solomon S.D., Louie E.K., Schiller N.B. (2010). Guidelines for the echocardiographic assessment of the right heart in adults: A report from the American Society of Echocardiography endorsed by the European Association of Echocardiography, a registered branch of the European Society of Cardiology, and the Canadian Society of Echocardiography. J. Am. Soc. Echocardiogr..

[35] Bossone E., D’andrea A., D’alto M., Citro R., Argiento P., Ferrara F., Cittadini A., Rubenfire M., Naeije R. (2013). Echocardiography in pulmonary arterial hypertension: From diagnosis to prognosis. J. Am. Soc. Echocardiogr..

[36] Sangkum L., Liu G.L., Yu L., Yan H., Kaye A.D., Liu H. (2016). Minimally invasive or noninvasive cardiac output measurement: An update. J. Anesth..

[37] Chemla D., Castelain V., Humbert M., Hébert J.L., Simonneau G., Lecarpentier Y., Hervé P. (2004). New formula for predicting mean pulmonary artery pressure using systolic pulmonary artery pressure. Chest.

[38] Fussner L.A., Krowka M.J. (2016). Current Approach to the Diagnosis and Management of Portopulmonary Hypertension. Curr. Gastroenterol. Rep..

[39] Horn P.S., Pesce A.J. (2005). Reference Intervals. A User’s Guide.

[40] Maslennikov R., Driga A., Ivashkin K., Ivashkin V. (2018). NT-proBNP as a biomarker for hyperdynamic circulation in decompensated cirrhosis. Gastroenterol. Hepatol. Bed Bench.

[41] Ferral H., Fimmel C.J., Sonnenberg A., Alonzo M.J., Aquisto T.M. (2021). Transjugular Liver Biopsy with Hemodynamic Evaluation: Correlation between Hepatic Venous Pressure Gradient and Histologic Diagnosis of Cirrhosis. J. Clin. Imaging Sci..

[42] Berg R.D., Garlington A.W. (1979). Translocation of certain indigenous bacteria from the gastrointestinal tract to the mesenteric lymph nodes and other organs in a gnotobiotic mouse model. Infect. Immun..

[43] Poxton I.R., Edmond D.M. (1995). Biological activity of Bacteroides lipopolysaccharide—Reappraisal. Clin. Infect. Dis..

[44] Maslennikov R., Ivashkin V., Efremova I., Poluektova E., Shirokova E. (2021). Probiotics in Hepatology: An Update. World J. Hepatol..

